# Evidence-based clinical practice guidelines for metabolic dysfunction-associated steatotic liver disease (MASLD) 2026

**DOI:** 10.1007/s00535-026-02408-2

**Published:** 2026-05-13

**Authors:** Norio Akuta, Tomomi Kogiso, Kenichi Ikejima, Motoyuki Otsuka, Takumi Kawaguchi, Miwa Kawanaka, Hirokazu Takahashi, Nobuharu Tamaki, Hayato Nakagawa, Hayato Hikita, Hideki Fujii, Kanji Yamaguchi, Masato Yoneda, Kazuyoshi Kon, Akira Uchiyama, Yuya Seko, Sadatsugu Sakane, Ryuta Shigefuku, Naoto Fujiwara, Michihiro Iwaki, Takashi Kobayashi, Takuya Adachi, Yasuto Takeuchi, Tsubasa Tsutsumi, Dan Nakano, Kaoru Shibayama, Noriyo Urata, Hisamitsu Miyaaki, Hidekatsu Kuroda, Masahiro Koseki, Hirohito Sone, Yasuhiro Matsubayashi, Keisuke Kakisaka, Atsushi Takai, Kazuo Notsumata, Masataka Seike, Yoshiyuki Takei, Yoshifumi Takeyama, Susumu Eguchi, Sumio Watanabe, Hajime Isomoto, Hiroshi Yotsuyanagi, Takao Itoi, Tetsuo Takehara, Satoshi Mochida

**Affiliations:** 1 Guidelines Committee for Creating and Evaluating the “Evidence-Based Clinical Practice Guidelines for Metabolic Dysfunction-Associated Steatotic Liver Disease 2026 (3rd Edition)”, The Japanese Society of Gastroenterology/The Japan Society of Hepatology, 6 F Shimbashi i-MARK Building, 2-6-2 Shimbashi, Minato-ku, Tokyo, 105-0004 Japan; 2 The Japan Society of Hepatology, Kashiwaya 2 Building 5 F, 3-28-10 Hongo, Bunkyo-ku, Tokyo, 113-0033 Japan; 3 Department of Hepatology, Toranomon Hospital, 2-2-2, Toranomon, Minato-ku, Tokyo, 105-8470 Japan; 4 Institute of Gastroenterology, Department of Internal Medicine, Tokyo Women’s Medical University, 8-1, Kawada-cho, Shinjuku-ku, Tokyo, 162-8666 Japan

**Keywords:** MASLD guidelines, Cardiometabolic risk factors, Noninvasive liver disease assessment, Treatment

## Abstract

Metabolic dysfunction-associated steatotic liver disease (MASLD) has emerged as the most prevalent chronic liver disease worldwide, with an increasing number of patients progressing to cirrhosis and hepatocellular carcinoma (HCC). Early identification of individuals at high risk for advanced fibrosis is essential for preventing liver-related events and improving long-term outcomes. This guideline summarizes current evidence and provides practical recommendations for the diagnosis, risk stratification, and management of MASLD in clinical practice. These recommendations were developed through systematic evaluation of the literature and expert consensus. Diagnostic approaches, including noninvasive fibrosis assessment tools, imaging, biochemical testing, and indications for referral to gastroenterology/hepatology specialists, were reviewed. Advanced fibrosis is the major determinant of liver-related morbidity and mortality in MASLD. The Fibrosis-4 (FIB-4) index is a widely accessible and reliable first-line noninvasive tool for fibrosis risk stratification. Patients with elevated FIB-4 values or clinical features suggestive of progressive liver injury should undergo secondary assessment with elastography and be referred to a specialist when appropriate. Liver biopsy is not routinely required for the diagnosis of MASLD but remains essential for establishing a definitive diagnosis of at-risk metabolic dysfunction-associated steatohepatitis (MASH), assessing inflammatory activity, resolving discrepancies between noninvasive tests, and differentiating MASLD from other chronic liver diseases. Lifestyle modification remains the foundation of management, with pharmacological therapy considered for patients with a high likelihood of at-risk MASH. MASLD represents a heterogeneous disease spectrum in which advanced fibrosis is the key prognostic factor. These guideline recommendations aim to standardize clinical practice and support evidence-based management of MASLD in Japan.

**Supplementary Information:**

The online version contains supplementary material available at 10.1007/s00535-026-02408-2.

## Introduction

### Concept and definition of metabolic dysfunction-associated steatotic liver disease (MASLD)

**Fig. 1:**
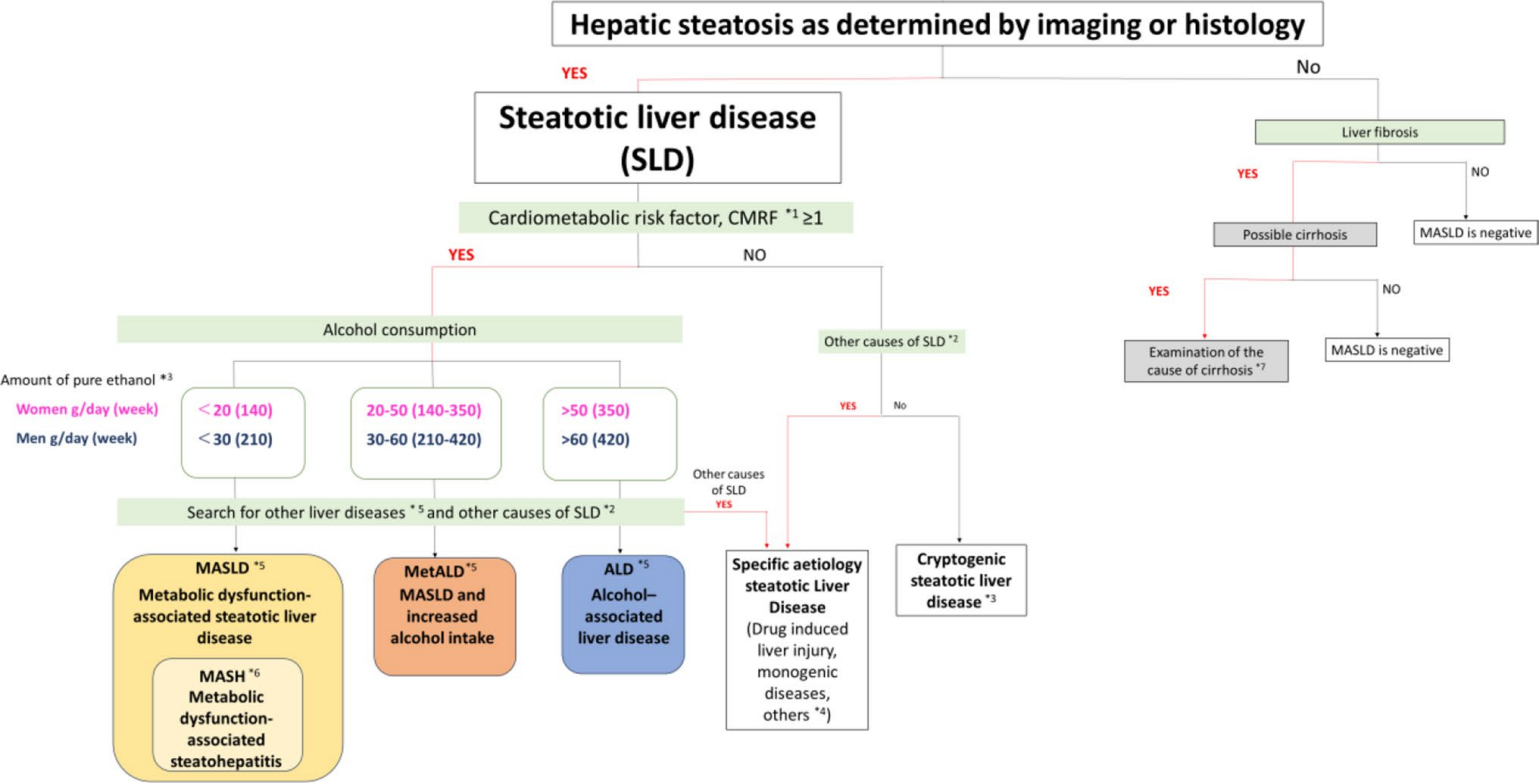
Diagnostic algorithm of SLD. MASLD is defined as SLD with hepatic steatosis affecting ≥ 5% of hepatocytes, as assessed by imaging or histology, in individuals with at least one CMRF (Table 1). Alcohol intake must be < 20 g/day in women and < 30 g/day in men, and other causes of steatosis should be excluded. *1. See Table 1. *2. See Table 2 for causes of SLD other than MASLD, MetALD, or ALD. *3. Liver injury may occur even with small amounts and short durations of alcohol consumption, particularly in women and individuals with reduced ALDH2 activity (see Epidemiology and natural history, FRQ). *4. Hepatitis C virus infection, particularly genotype 3, can induce hepatic steatosis (see Definitions and concepts). *5. Coexisting liver diseases may be present, such as MASLD with viral hepatitis or autoimmune liver diseases. *6. Cases showing histological features of steatosis, inflammation, and hepatocellular ballooning. Even in the absence of liver biopsy, MASH should be considered in patients with MASLD when NILDA suggests hepatocellular injury or progressive fibrosis. *7. In patients with CMRF, burned-out MASLD should be considered, and referral to a specialist is recommended. ALD, alcohol-associated liver disease; ALDH2, aldehyde dehydrogenase 2; CMRF, cardiometabolic risk factor; MASLD, metabolic dysfunction-associated steatotic liver disease; MASH, metabolic dysfunction-associated steatohepatitis; MetALD, MASLD with increased alcohol intake; NILDA, noninvasive liver disease assessment; SLD, steatotic liver disease

MASLD is a disease concept proposed in 2023 by an international consensus to replace the term nonalcoholic fatty liver disease (NAFLD) [1–3] (Fig. 1). MASLD provides a revised diagnostic framework that more accurately reflects the underlying metabolic pathophysiology. It is defined as steatotic liver disease (SLD) with hepatic steatosis affecting ≥ 5% of hepatocytes, as assessed by imaging or histology, in individuals with at least one cardiometabolic risk factor (CMRF) (Table 1). Alcohol intake must be < 20 g/day in women and < 30 g/day in men, and other causes of hepatic steatosis should be excluded.

**Table 1:** Diagnostic criteria for CMRFs

Variables	Adult criteria
Body mass index Waist circumference	≥ 23 kg/m^2^ (≥ 25 kg/m^2^ for non-Asian populations) Men: > 94 cm, women: > 80 cm
Fasting glucose 2-h plasma glucose HbA_1C_ Type 2 diabetes or treatment	≥ 100 mg/dL ≥ 140 mg/dL ≥ 5.7%
Blood pressure or treatment for hypertension	≥ 130/85 mmHg
Plasma triglycerides or lipid-lowering treatment	≥ 150 mg/dL
HDL-cholesterol or lipid-lowering treatment	Men: ≤ 40 mg/dL, women: ≤ 50 mg/dL

CMRF, cardiometabolic risk factor; HDL, high-density lipoprotein

MASLD is strongly associated with obesity, insulin resistance, dyslipidemia, and hypertension, and it may progress to fibrosis, cirrhosis, and hepatocellular carcinoma (HCC). It is also recognized as a systemic disease associated with increased risks of cardiovascular disease (CVD), chronic kidney disease (CKD), and extrahepatic malignancies. When steatosis is accompanied by inflammation and hepatocellular ballooning, the condition is termed metabolic dysfunction-associated steatohepatitis (MASH). The MASLD/MASH concept replaces the stigmatizing terminology of NAFLD/nonalcoholic steatohepatitis (NASH) while maintaining essentially the same clinical features and diagnostic algorithms; therefore, evidence accumulated under NAFLD/NASH remains applicable [4, 5].

Following the publication of the 2014 guidelines and their 2020 revision, accumulating evidence has established hepatic fibrosis as a key determinant of prognosis in MASLD. This third revision emphasizes the MASLD concept, noninvasive liver disease assessment, and updated evidence-based treatment recommendations, with additional clinical questions incorporated [6]. Evidence levels A, B, and C correspond to high, moderate, and low quality of evidence, respectively, according to the Grades of Recommendation Assessment, Development and Evaluation (GRADE) framework (The GRADE Working Group. Grading quality of evidence and strength of recommendations. *BMJ* 2004;328:1490–1494).

### Diagnostic flowchart for SLD

This diagnostic flowchart outlines a stepwise approach to the diagnosis of SLD (Fig. 1). First, the presence of hepatic steatosis is confirmed by imaging studies or histological examination. Second, in patients with hepatic steatosis, the presence of at least one CMRF is assessed to determine whether the condition meets the criteria for MASLD. Third, alcohol consumption is evaluated to classify patients as having MASLD, MASLD with increased alcohol intake (MetALD), or alcohol-associated liver disease (ALD), according to predefined alcohol intake thresholds. In parallel, other causes of hepatic steatosis should be systematically excluded before establishing the final diagnosis (Table 2).

**Table 2:** Specific etiologies of SLD other than MASLD, MetALD, and ALD

Causes	Features/specific etiologies
HCV infection	Especially genotype 3
Drug-induced liver injury	Corticosteroids, tamoxifen, amiodarone, irinotecan, methotrexate, lomitapide, sodium valproate, fluorouracil, etc
Pregnancy	Acute fatty liver of pregnancy, HELLP syndrome
Wilson’s disease	Early onset, neuropsychiatric symptoms, low ceruloplasmin
Nutrition deficiency/malnutrition	SLD due to nutritional deficiency, weight loss, metabolic and bariatric surgery, anorexia nervosa, pancreatic resection, malabsorption
Endocrine disorders	Hypothyroidism, polycystic ovary syndrome, growth hormone deficiency, panhypopituitarism (primary or secondary)

Other conditions that have been reported include hypobetalipoproteinemia, lipodystrophy, lysosomal acid lipase deficiency (Wolman disease, cholesteryl ester storage disease), and celiac disease, but these are extremely rare in the Japanese population

ALD, alcohol-associated liver disease; HCV, hepatitis C virus; HELLP syndrome, hemolysis, elevated liver enzymes, and low platelets syndrome; MASLD, metabolic dysfunction-associated steatotic liver disease; MetALD, MASLD with increased alcohol intake; SLD, steatotic liver disease

When steatosis is absent, alternative etiologies of liver disease should be explored, particularly in patients with metabolic risk factors who may have “burned-out” MASLD. If advanced fibrosis or cirrhosis is suspected, further etiological evaluation is required, and referral to a gastroenterology/hepatology specialist is recommended.

Consistent evidence indicates that even modest alcohol intake is associated with fibrosis progression [7–9], synergistic increases in CVD risk [10], and higher mortality in MASLD [11]. These findings suggest that no safe threshold of alcohol intake exists for patients with MASLD. In Japan, hepatotoxicity may occur at lower intake levels, particularly in women and individuals with aldehyde dehydrogenase 2 deficiency [12, 13], underscoring the need for refined thresholds and harmonized criteria for MetALD.

## Epidemiology

The prevalence of MASLD varies across countries and regions and is generally higher in men than in women. According to a meta-analysis of global adult populations, the pooled prevalence of MASLD is approximately 30% [14].

In Japan, a systematic review and meta-analysis by Ito et al. [15] reported a prevalence of 25.5% (95% confidence interval [CI]: 23.3–27.9). Temporal trends showed an increase from 22.2% in 1984–2005 to 29.6% in 2011–2016, suggesting a rising prevalence over time. A consistent sex difference has been observed; Riazi et al. [16] reported prevalence rates of approximately 40% in men and 26% in women, findings that align with Japanese health check-up cohort data (37.4% in men and 18.1% in women) [17].

Evidence regarding the prevalence of MASLD in children remains limited. However, a meta-analysis published in 2024 estimated the global pediatric prevalence to be 13% (95% CI: 9–18) [18]. By contrast, the prevalence of MASH remains uncertain because of declining liver biopsy rates and potential selection bias in histology-based studies.

### BQ. Is the risk of liver-related events increased in MASLD?


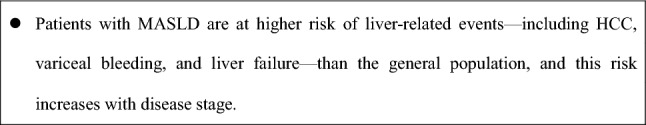


Patients with MASLD exhibit an increased risk of liver-related events compared with the general population. A meta-analysis of 36 observational studies including 24,894 individuals, incorporating Japanese cohorts, reported fibrosis progression rates of 6.5, 6.9, 5.8, and 4.5 cases per 100 person-years for transitions from F0 to F1, F1 to F2, F2 to F3, and F3 to F4, respectively [19]. Among patients with compensated cirrhosis, the annual incidence of decompensation events is approximately 8% [20]. Although disease progression varies substantially among individuals, the average time from F0–1 to decompensated cirrhosis has been estimated at 30–35 years [21]. In a Swedish population-based cohort, biopsy-confirmed MASLD was associated with a 17-fold higher risk of HCC compared with matched controls (adjusted hazard ratio [HR] 17.08) [22].

Similarly, a multicenter Japanese cohort of 1398 biopsy-proven cases reported liver-related event rates of 2.8, 5.4, 21.5, and 90.1 per 1000 person-years for fibrosis stages F0–1, F2, F3, and F4, respectively, with corresponding HCC incidence rates of 1.7, 4.5, 14.2, and 16.9 per 1,000 person-years [23]. Advanced age, type 2 diabetes (T2DM), and a family history of cirrhosis were identified as significant predictors of adverse outcomes [23–25].

Additionally, so-called “burned-out” MASLD—characterized by low hepatic fat content despite advanced fibrosis—is associated with a poorer prognosis [26]. A prospective study using FibroScan^®^ demonstrated that patients with low-grade steatosis (grade ≤ 1) had significantly higher rates of liver-related events and hepatic failure than those with moderate or severe steatosis [27]. These findings indicate that a reduction in hepatic fat content does not necessarily reflect disease regression.

### BQ. Is there an increased risk of non-liver-related events in MASLD?


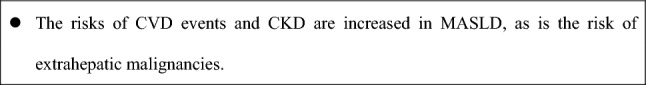


**Fig. 2:**
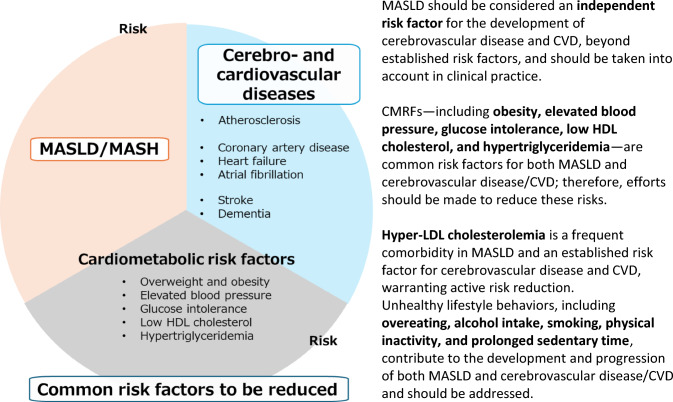
Association of MASLD/MASH with CMRF and CVD risk. MASLD should be considered an independent risk factor for the development of cerebrovascular disease and CVD, beyond established risk factors, and should be taken into account in clinical practice. CMRFs—including obesity, elevated blood pressure, glucose intolerance, low HDL cholesterol, and hypertriglyceridemia—are common risk factors for both MASLD and cerebrovascular disease/CVD; therefore, efforts should be made to reduce these risks. Hyper-LDL cholesterolemia is a frequent comorbidity in MASLD and an established risk factor for cerebrovascular disease and CVD, warranting active risk reduction. Unhealthy lifestyle behaviors, including overeating, alcohol intake, smoking, physical inactivity, and prolonged sedentary time, contribute to the development and progression of both MASLD and cerebrovascular disease/CVD and should be addressed. ALD, alcohol-associated liver disease; CMRF, cardiometabolic risk factor; CVD, cardiovascular disease; HDL, high-density lipoprotein; LDL, low-density lipoprotein; MASLD, metabolic dysfunction-associated steatotic liver disease; MASH, metabolic dysfunction-associated steatohepatitis; MetALD, MASLD with increased alcohol intake

Beyond hepatic outcomes, MASLD confers increased risks of CVD, CKD, and extrahepatic malignancies (Fig. 2). A meta-analysis including approximately 5.8 million participants demonstrated a 45% higher risk of CVD in individuals with MASLD (HR 1.45) [28]. Similarly, a Japanese cohort of 2.9 million individuals showed a 35% higher risk of CVD events in patients with MASLD who had a body mass index (BMI) of ≥ 23 kg/m^2^ than in controls without liver disease [29]. Meta-analyses have also revealed that MASLD is associated with a 1.45- to 1.53-fold increased risk of incident CKD [30, 31].

MASLD is further associated with an increased risk of extrahepatic malignancies, particularly colorectal cancer. In a Japanese cohort of approximately 6 million individuals, patients with MASLD had a higher risk of colorectal cancer (HR 1.28) [32]. Elevated risks of gastric, esophageal, and gynecologic cancers have also been reported [33, 34]. In a Japanese study of 1,398 patients with biopsy-confirmed MASLD, 31 developed HCC, while 31 experienced CVD events and 68 developed extrahepatic malignancies—including breast, gastric, and colorectal cancers—during follow-up [23].

## Clinically significant differences between MASLD and MetALD

Consistent evidence indicates that MetALD carries a higher risk of liver-related events than MASLD [35]. In a large Japanese cohort including approximately 760,000 individuals, the risk of liver-related events was significantly higher in MetALD than in MASLD (HR 1.42) [35]. In the same cohort, the risk of CVD was lower in MetALD than in MASLD (HR 0.68), suggesting a higher CVD risk in MASLD [35]. Conversely, a prospective cohort study of approximately 9,000 individuals reported that compared with the non-SLD group, the HR for CVD events was 1.27 in MASLD and 1.88 in MetALD, suggesting a higher CVD risk in MetALD [36]. Further studies are warranted to clarify the differential CVD risk between MASLD and MetALD.

## Pathogenesis and genomic background

Regarding the pathogenesis of MASLD, excessive accumulation of free fatty acids—particularly saturated lipids—induces mitochondrial dysfunction and reactive oxygen species generation, leading to hepatocyte apoptosis and necroinflammation [37–39]. Endoplasmic reticulum stress and activation of the unfolded protein response promote c-Jun N-terminal kinase (JNK) signaling, further contributing to cell death [40]. Impaired autophagy also plays a role by promoting lipid droplet accumulation and defective organelle clearance [40, 41]. Crosstalk among hepatocytes, Kupffer cells, and hepatic stellate cells amplifies inflammation and fibrogenesis through cytokines such as tumor necrosis factor-α and transforming growth factor-β.

Genetic factors have also been shown to contribute to the onset and progression of MASLD [42].

### FRQ. How do genetic factors contribute to MASLD development and progression?


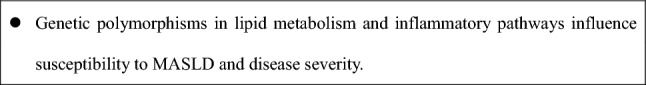


Multiple genetic polymorphisms, including those in patatin-like phospholipase domain-containing protein 3 (*PNPLA3*), transmembrane 6 superfamily member 2 (*TM6SF2*), hydroxysteroid 17-beta dehydrogenase 13 (*HSD17B13*), and membrane-bound O-acyltransferase domain-containing protein 7 (*MBOAT7*), affect disease severity and prognosis [42]. The *PNPLA3* I148M variant is particularly prevalent in East Asian populations and confers an approximately twofold increased risk of advanced fibrosis [43]. The *HSD17B13* loss-of-function variant attenuates inflammation and fibrosis and is considered a protective allele [44].

## Diagnosis and risk stratification by noninvasive liver disease assessment

### CQ. Are biomarkers and scoring systems useful for diagnosing and assessing fibrosis and inflammation in MASLD? (Table 3)


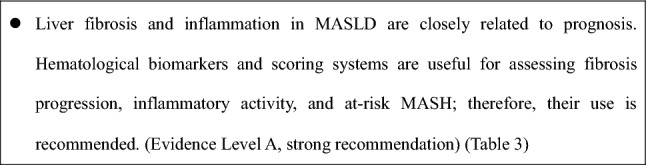


Fibrosis stage is the strongest predictor of prognosis in MASLD/MASH, making accurate assessment essential. Combining biomarkers and scoring systems enables reliable fibrosis staging. Type IV collagen 7S measured by chemiluminescent enzyme immunoassay shows improved diagnostic accuracy, with proposed cutoffs of 3.8 ng/mL for F2 [45]. Reported enhanced liver fibrosis (ELF) test thresholds are approximately 9.2–9.8 for F2, 9.8 for F3, and 11.8 for F4 [46, 47]. Although the assay for Mac-2-binding protein glycosylation isomer (M2BPGi) has been updated to a quantitative M2BPGi (M2BPGi-Qt) method with improved analytical precision, evidence from Japan remains limited. The proposed cutoff for significant fibrosis (≥ F2) is ≥ 1.0 C.O.I. [48, 49], while autotaxin values vary by sex [50].

**Table 3:** Cutoff values for fibrosis staging using NILDA modalities [1, 47, 61, 114, 115]

	VCTE	MRE	SWE	ELF
≥ F2	≥ 8 kPa	≥ 3.14 kPa	≥ 8 kPa (≥ 1.6 m/s)*	9.2–9.8**

ELF, enhanced liver fibrosis; MRE, magnetic resonance elastography; NILDA, noninvasive liver disease assessment; SWE, shear-wave elastography; VCTE, vibration-controlled transient elastography

*Cutoff values for SWE may vary depending on device type and acquisition parameters; reference values specific to each system should be considered

**Reported ELF cutoff values vary across studies; therefore, a representative range is shown

Cytokeratin 18 fragments (CK-18F), which are already reimbursed in Japan, reflect disease activity and may help identify at-risk MASH when CK-18F levels are ≥ 200–260 U/L are combined with a Fibrosis-4 (FIB-4) score of > 1.3 [51].

### CQ. Are noninvasive steatosis diagnostic methods useful for diagnosing SLD?


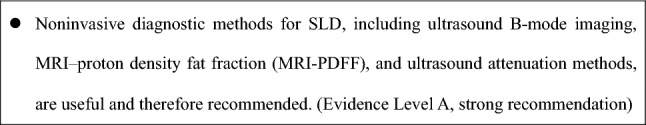


Ultrasound B-mode imaging has traditionally been used for screening and initial evaluation of hepatic steatosis, based on findings such as increased liver–kidney contrast and posterior beam attenuation [52]. Because of its noninvasiveness and convenience, it remains widely used; however, its sensitivity is limited for mild steatosis (< 20–30%), and it is subject to operator dependency and limited objectivity [53].

To overcome these limitations, several noninvasive quantitative techniques have been developed. Among them, MRI-PDFF shows the strongest correlation with histologic fat content and is considered a reliable quantitative method. A Japanese study demonstrated excellent diagnostic performance, with an area under the receiver operating characteristic curve (AUROC) of 0.90 for detecting grade ≥ 2 steatosis [54]. MRI-PDFF is being increasingly used as a reference standard in clinical studies in place of liver biopsy.

Ultrasound-based attenuation techniques—including controlled attenuation parameter, attenuation imaging, ultrasound-guided attenuation parameter, attenuation measurement, and ultrasound-derived fat fraction—have also been incorporated into clinical practice. Controlled attenuation parameter, attenuation imaging, ultrasound-guided attenuation parameter, and attenuation measurement are currently reimbursed in Japan [55–59]. These techniques provide better diagnostic performance than conventional B-mode imaging and can be performed easily in routine settings.

### CQ. Is hepatic fat quantification useful for predicting progression of liver disease?


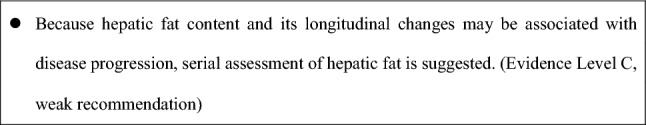


### CQ. Is ultrasound elastography useful for diagnosing fibrosis in MASLD?


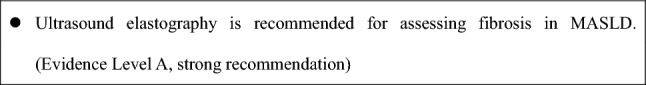


Ultrasound elastography is a noninvasive technique that quantifies tissue stiffness by analyzing the propagation of ultrasound-induced shear waves. The main modalities include vibration-controlled transient elastography (VCTE) (FibroScan®), two-dimensional shear-wave elastography (2D-SWE), and point shear-wave elastography (p-SWE).

VCTE provides validated cutoffs for fibrosis staging. Meta-analyses report AUROCs of approximately 0.83 for ≥ F2, 0.85 for ≥ F3, and 0.89 for F4 [60]. Thresholds of 8 kPa (rule-out) and 12 kPa (rule-in) have been proposed for F3 fibrosis, and liver stiffness measurements of > 10 kPa are associated with an increased risk of HCC [61]. In 2025, the U.S. Food and Drug Administration accepted FibroScan® liver stiffness measurement as a reasonable alternative to liver biopsy in clinical trials for noncirrhotic MASH.

Two-dimensional SWE allows real-time visualization of shear-wave propagation and shows AUROCs ranging from 0.72 to 0.88 for fibrosis staging [60]. Suggested cutoffs for F3 are approximately 8 kPa (rule-out) and 10.5 kPa (rule-in). p-SWE [61], which measures stiffness at a single site, also demonstrates high diagnostic accuracy, with AUROCs of 0.86–0.90 for fibrosis stages ≥ F2–F4 [60].

Large international cohort studies, including one involving more than 16,000 patients with MASLD, have shown that VCTE predicts liver-related outcomes comparably to—or better than—liver biopsy, with longitudinal changes further improving prognostic accuracy [62]. These findings support the role of ultrasound elastography as a practical alternative to liver biopsy.

### CQ. Is magnetic resonance elastography (MRE) useful for diagnosing fibrosis in MASLD?


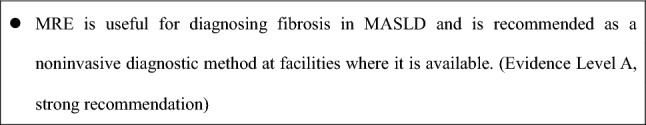


## Risk stratification and follow-up flowchart for high-risk liver disease

**Fig. 3:**
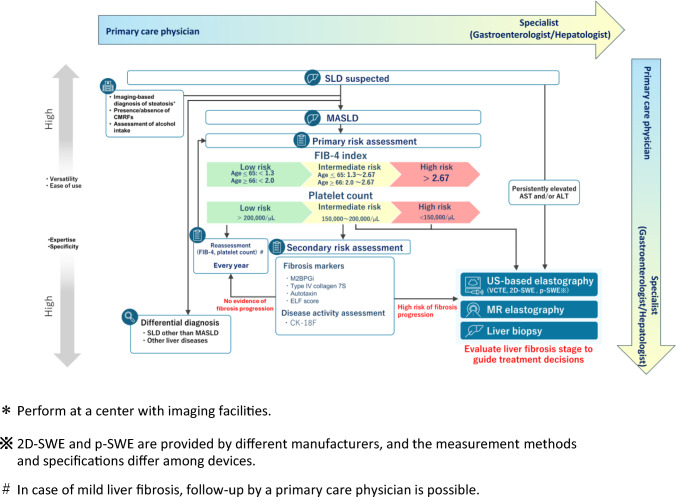
Risk stratification and follow-up flowchart for high-risk liver disease. This flowchart presents a stepwise approach for identifying and managing patients with MASLD. Evaluation is designed as a shared-care model between primary care physicians and gastroenterology/hepatology specialists, combining accessible screening tools with specialized diagnostic modalities. The process begins with identification of suspected SLD, including MASLD, based on clinical history, alcohol intake assessment, metabolic risk factors, and imaging findings suggestive of hepatic steatosis. In facilities capable of imaging-based fat quantification, ultrasound or alternative imaging modalities are recommended to confirm steatosis. First-line risk assessment is based on the FIB-4 index and platelet count. Patients meeting any of the following three criteria should be referred to a gastroenterologist/hepatologist for further evaluation: elevated fibrosis or disease progression markers, intermediate risk based on FIB-4 index and/or low platelet count, or persistently elevated AST or ALT levels. ALT, alanine aminotransferase; AST, aspartate aminotransferase; FIB-4, Fibrosis-4 index; MASLD, metabolic dysfunction-associated steatotic liver disease; SLD, steatotic liver disease

The framework shown in Fig. 3 adopts a shared-care continuum in which primary care physicians and gastroenterology/hepatology specialists collaboratively and sequentially contribute to MASLD management according to disease severity. The process begins with identifying suspected SLD based on clinical history, assessment of alcohol intake, metabolic risk factors, and imaging findings suggestive of hepatic steatosis. In facilities capable of imaging-based fat quantification, ultrasound or alternative imaging modalities are recommended to confirm steatosis.

First-line risk assessment is based on the FIB-4 index and platelet count. The FIB-4 index is a noninvasive fibrosis score calculated from age, platelet count, aspartate aminotransferase (AST), and alanine aminotransferase (ALT), and it is useful for fibrosis screening [61, 63]. Referral algorithms for MASLD have been proposed in several guidelines [2, 61, 64, 65]. All of these algorithms use the FIB-4 index as the primary risk stratification tool and recommend referral to a gastroenterologist/hepatologist when FIB-4 is > 2.67 or when FIB-4 is 1.3–2.67 (≥ 2.0 in individuals aged ≥ 66 years). Meta-analyses report AUROC values ranging from 0.65 to 0.76 [66, 67]. The platelet count provides complementary risk stratification because lower counts may reflect more advanced fibrosis (low risk: > 200,000/µL; intermediate risk: 150,000–200,000/µL; high risk: < 150,000/µL).

In Japan, several serum fibrosis markers are available [68], including type IV collagen 7S [45], M2BPGi [48], autotaxin [50] and the ELF test [46, 47], which incorporates hyaluronic acid, tissue inhibitor of metalloproteinase 1 (TIMP-1), and procollagen III aminoterminal peptide (P-III-P). These markers provide additional information for refining fibrosis staging and assessing inflammatory activity, thereby helping determine the need for specialist referral.

Liver biopsy is reserved for cases in which noninvasive tools yield inconclusive results or when a definitive diagnosis is required to guide treatment decisions. Low-risk patients, particularly those with minimal fibrosis, may continue follow-up in primary care with periodic reassessment, typically once per year. Similarly, patients with mild liver fibrosis may be managed by a primary care physician.

Overall, this structured approach improves the accuracy of identifying high-risk liver disease and supports efficient, collaborative care between primary care physicians and liver specialists.

### BQ. Is a liver biopsy required for the diagnosis of MASLD?


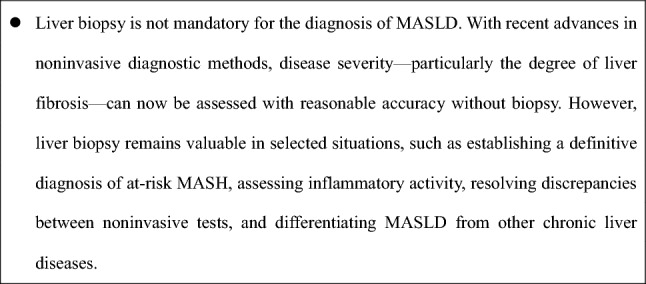


## Criteria for gastroenterological consultation in patients with MASLD

Cirrhosis and HCC are major determinants of prognosis in MASLD. Accordingly, patients with advanced liver fibrosis, who are at higher risk for these outcomes, should be referred to a gastroenterologist/hepatologist. Referral is recommended for patients meeting any of the following three criteria (Table 4): elevated fibrosis markers or evidence of disease progression, intermediate risk based on the FIB-4 index and/or low platelet count, or persistently elevated AST or ALT levels. To improve diagnostic accuracy, VCTE, 2D-SWE, p-SWE, and MRE are recommended for evaluating fibrosis progression and informing treatment strategies.

**Table 4:** Criteria for referral to gastroenterology/hepatology in patients with MASLD

FIB-4 index > 2.67 FIB-4 index 1.3–2.67 (≥ 2.0 in individuals aged ≥ 66 years) and any NILDA result above the cutoff, including: i) VCTE ii) MRE iii) SWE iv) Serum biomarkers (ELF, type IV collagen 7S, M2BPGi, or autotaxin)
Platelet count < 200,000/µL Persistently elevated AST and/or ALT levels Imaging findings suggestive of cirrhosis

ALT, alanine aminotransferase; AST, aspartate aminotransferase; ELF, enhanced liver fibrosis; FIB-4, Fibrosis-4; MASLD, metabolic dysfunction-associated steatotic liver disease; M2BPGi, Mac-2-binding protein glycosylation isomer; MRE, magnetic resonance elastography; NILDA, noninvasive liver disease assessment; SWE, shear-wave elastography; VCTE, vibration-controlled transient elastography

### FRQ. What is the appropriate screening method for liver cirrhosis and liver cancer due to MASLD?


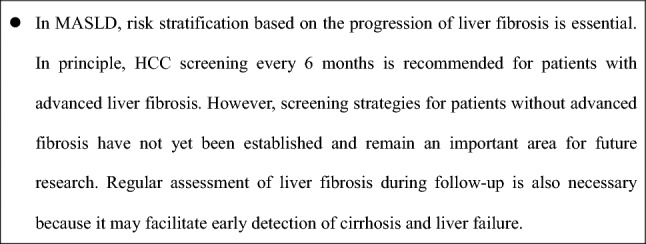


## Follow-up and management of CVD and extrahepatic malignancies

Clear criteria for CVD screening and specialist referral have not been established. Nevertheless, MASLD is associated with an increased risk of CVD, and both MASLD itself and disease progression may contribute to this risk. Active screening and referral to appropriate specialists are therefore generally recommended (Fig. 3). Shared risk factors for MASLD and CVD include obesity, elevated blood pressure, impaired glucose tolerance, low high-density lipoprotein cholesterol levels, and hypertriglyceridemia, and efforts should be made to mitigate these risks. Elevated low-density lipoprotein cholesterol is also a common CVD risk factor, and appropriate lipid-lowering strategies should be implemented in patients with MASLD.

With respect to extrahepatic malignancies, age-appropriate screening for colorectal cancer and other malignancies is suggested. However, optimal screening methods and intervals have not yet been adequately validated.

## Treatment

**Fig. 4:**
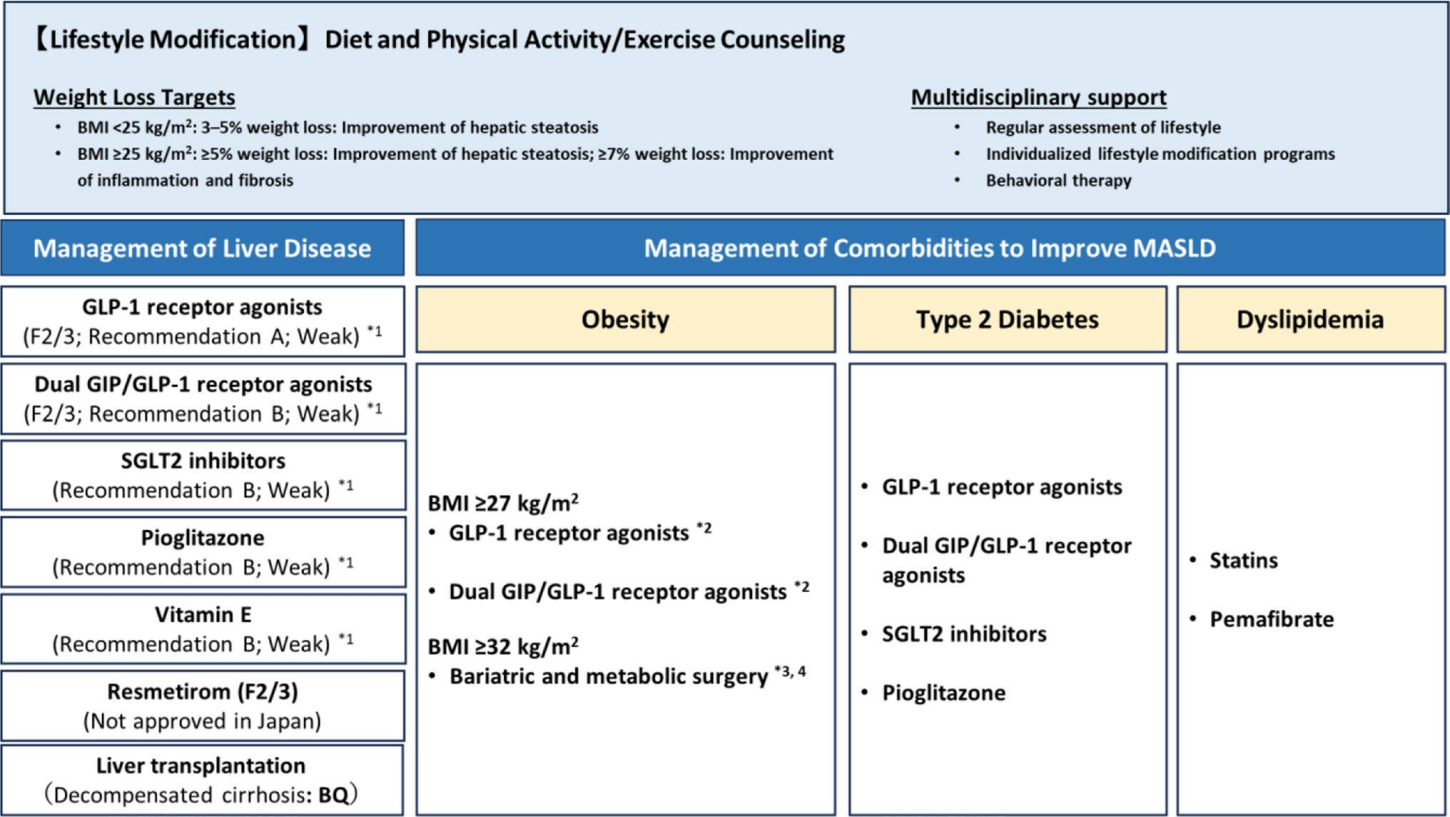
Management strategies for patients with MASLD. (The information presented in this figure reflects the clinical and regulatory situation in Japan as of 23 September 2025.) *1. GLP-1RA, dual GIP/GLP-1RA, SGLT2 inhibitors, pioglitazone, and vitamin E are not covered by insurance for MASLD in Japan. *2. Prescription by physicians experienced in obesity management is recommended. Treatment is indicated for patients with MASLD and hypertension, dyslipidemia, or T2DM who have not achieved sufficient effects with dietary and exercise therapy and who meet one of the following criteria: BMI ≥ 27 kg/m^2^ with at least one obesity-related comorbidity. BMI ≥ 35 kg/m^2^. *3. Treatment is indicated for patients with MASLD who have not achieved weight loss despite ≥ 6 months of medical therapy and who meet one of the following criteria: BMI 32.0–34.9 kg/m^2^ with at least one comorbidity (T2DM with HbA1c ≥ 8.0%, hypertension, dyslipidemia, or obstructive sleep apnea), BMI ≥ 35 kg/m^2^. *4. In patients with compensated liver cirrhosis, surgical indications should be evaluated with particular caution. BMI, body mass index; GIP, glucose-dependent insulinotropic polypeptide; GLP-1RA, glucagon-like peptide-1 receptor agonist; HbA1c, hemoglobin A1c; MASLD, metabolic dysfunction-associated steatotic liver disease; SGLT2, sodium–glucose cotransporter 2; T2DM, type 2 diabetes mellitus

Lifestyle modification is fundamental to the management of MASLD and should be maintained even during pharmacologic therapy [69] (Fig. 4). Weight loss, reduced energy intake, and appropriate nutritional balance are effective in improving MASLD [70–73]. Weight loss of ≥ 5% improves hepatic steatosis, while ≥ 10% may lead to fibrosis regression [26, 74–76]. In non-obese individuals, even a 3–5% reduction in body weight may be beneficial [77]. Calorie-restricted diets and balanced dietary patterns, such as the Mediterranean or Japanese diet, improve insulin sensitivity and reduce intrahepatic fat [78–80].

Exercise is beneficial not only for reducing intrahepatic fat but also for improving glucose and lipid metabolism and mitigating sarcopenia, and it is considered a core component of treatment. Aerobic and resistance exercise totaling ≥ 150 min per week improves steatosis and cardiometabolic parameters, even in the absence of significant weight loss [81, 82]. As of 2025, no pharmacologic therapy had been approved for MASLD in Japan; however, several agents are under investigation in clinical trials.

## Pharmacological treatments

At-risk MASH is defined as an NAFLD activity score of ≥ 4 with fibrosis stage of ≥ F2. This phenotype is associated with a higher risk of disease progression and represents a key target for pharmacological therapy.

### CQ. Are glucagon-like peptide-1 receptor agonists (GLP-1RA) effective for MASLD?


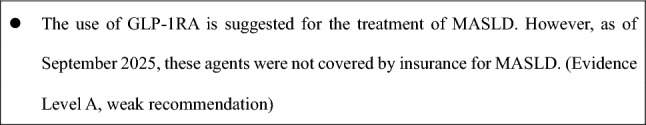


(a) GLP-1RA

GLP-1RA reduces hepatic fat by promoting weight loss, improving insulin sensitivity, and exerting direct anti-inflammatory and antifibrotic effects on the liver. In the LEAN trial, liraglutide achieved MASH resolution in 39% of patients [83]. Semaglutide, evaluated in the ESSENCE trial (2025), achieved MASH resolution in 63% of patients and fibrosis improvement in 37% [84, 85]. In 2025, the U.S. Food and Drug Administration granted accelerated approval of semaglutide for the treatment of MASH. Real-world data also indicate that GLP-1RA use is associated with a reduced risk of cirrhosis and liver-related mortality [86].

### CQ. Are glucose-dependent insulinotropic polypeptide (GIP)/GLP-1RA effective for MASLD?


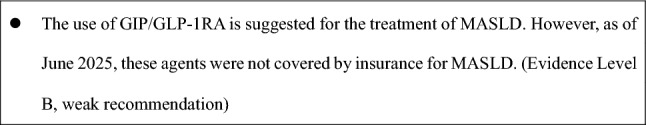


(b) Dual GIP/GLP-1RA

Tirzepatide combines GIP- and GLP-1-mediated actions, resulting in greater weight loss and broader metabolic benefits. In the SYNERGY-MASH trial, tirzepatide 15 mg achieved MASH resolution in 62% of patients and fibrosis improvement in 51% after 52 weeks [87]. The most common adverse events were gastrointestinal symptoms; these were generally mild to moderate, and overall tolerability was favorable.

### CQ. Are sodium–glucose cotransporter 2 (SGLT2) inhibitors effective for MASLD?


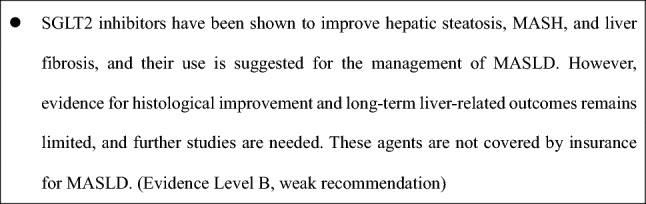


(c) SGLT2 inhibitors

SGLT2 inhibitors promote glycosuria and energy loss, leading to weight reduction and improved insulin resistance. Several randomized controlled trials, including the DEAN trial, have demonstrated significant improvements in hepatic steatosis, hepatocellular ballooning, and fibrosis [88–91]. Meta-analyses have confirmed improvements in ALT levels, liver stiffness, and fibrosis markers [92, 93]. In addition, SGLT2 inhibitors reduce cardiovascular and renal events, making them particularly attractive for patients with MASLD and T2DM [94].

### CQ. Is pioglitazone effective for MASLD?


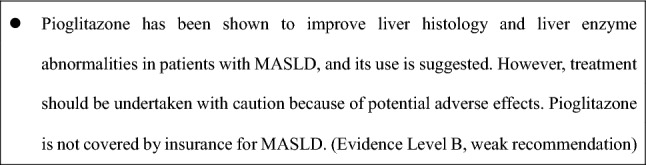


(d) Pioglitazone

As a peroxisome proliferator-activated receptor (PPAR)γ agonist, pioglitazone improves insulin sensitivity and reduces hepatic inflammation [95, 96]. Meta-analyses have demonstrated significant histological improvement, including improvements in ballooning and steatosis, in both diabetic and non-diabetic patients with MASH [97]. However, adverse effects such as weight gain, edema, heart failure, and osteoporosis limit its use, and caution is particularly warranted in elderly patients and those with heart failure [61].

### CQ. Are lipid metabolism-modifying agents effective for MASLD?

#### Pemafibrate


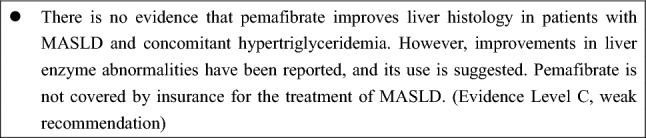


#### Statins


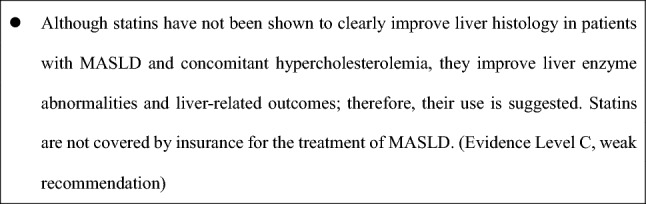


(e) Lipid-modifying agents

Pemafibrate, a selective PPARα modulator, reduces transaminase levels and fibrosis markers but lacks evidence of histological efficacy. It may be beneficial in patients with MASLD and hypertriglyceridemia [98].

Statins reduce cardiovascular events and may lower ALT levels and fibrosis risk [99, 100]. Observational studies have reported a decreased incidence of HCC and a reduced risk of liver-related events in patients with MASLD-related cirrhosis [101]. Accordingly, statins are recommended for patients with MASLD and dyslipidemia when clinically indicated.

Ezetimibe and omega-3 fatty acids lack sufficient evidence of efficacy [102–104] and are not recommended as MASLD-specific treatments.

(f) Hepatoprotective agents

Agents such as ursodeoxycholic acid and glycyrrhizin derivatives have not demonstrated consistent histological or biochemical benefits and are not recommended in current guidelines.

### CQ. Is vitamin E effective for MASLD?


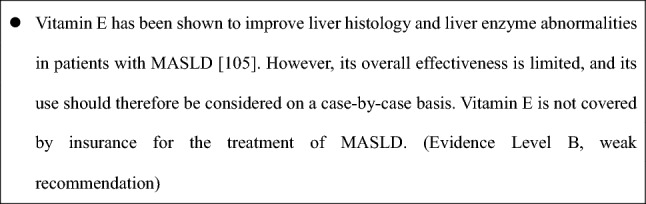


## Surgical treatment

In patients with MASLD and severe obesity, metabolic and bariatric surgery represents a useful treatment option for improving liver pathology. However, a comprehensive treatment strategy is required, taking into account surgical indications in patients with liver cirrhosis and the potential for postoperative recurrence of MASLD [106].

The number of liver transplants performed for decompensated cirrhosis due to MASLD is increasing. Although post-transplant survival is comparable to that observed in other liver diseases [107], the incidence of comorbidities, particularly CVD, remains high.

## Comprehensive management

Management of MASLD should be individualized, with lifestyle modification as the therapeutic foundation, pharmacologic therapy considered for high-risk or biopsy-proven MASH, and appropriate control of cardiovascular risk factors. Multidisciplinary care involving gastroenterologists/hepatologists, endocrinologists, and cardiologists is essential for improving long-term outcomes. The cost-effectiveness of liver cancer screening remains unclear in patients without cirrhosis. In addition, differences in health insurance systems between Japan and other countries may influence perspectives on the cost-effectiveness and implementation of screening strategies.

## Other candidate drugs

Several global phase 3 trials are currently evaluating novel agents targeting lipotoxicity, inflammation, and fibrosis. These include farnesoid X receptor agonists [108] (e.g., obeticholic acid), thyroid hormone receptor-β agonists [109] (e.g., resmetirom), and pan-PPAR agonists [110] (e.g., lanifibranor). In addition, several fibroblast growth factor 21 analogues [111, 112], such as efruxifermin and pegozafermin, are advancing through clinical development and are considered promising candidates for MASLD treatment. Combination strategies integrating metabolic and antifibrotic approaches are expected to further refine MASLD management, and gene-targeted therapies may enable future personalized treatment strategies [113].

### FRQ. Is resmetirom effective for MASH?


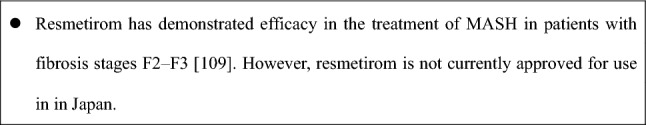


## Supplementary Information

Below is the link to the electronic supplementary material.

Supplementary file1 (DOCX 26 KB)

## Appendix

The members of the Guidelines Committee who created and evaluated. The Japanese Society of Gastroenterology (JSGE) and the Japanese Society of Hepatology (JSH) “Evidence-based Clinical Practice Guidelines for Metabolic Dysfunction-Associated Steatotic Liver Disease 2026 (3rd Edition)” are listed below.

### Creation Committee

Chair: Norio Akuta (Department of Hepatology, Toranomon Hospital). Vice-chair: Tomomi Kogiso (Institute of Gastroenterology, Department of Internal Medicine, Tokyo Women’s Medical University). Members: Kenichi Ikejima (Department of Gastroenterology, Juntendo University Graduate School of Medicine), Motoyuki Otsuka (Department of Gastroenterology and Hepatology, Academic Fields of Medicine, Dentistry, and Pharmaceutical Sciences, Okayama University), Takumi Kawaguchi (Division of Gastroenterology, Department of Medicine, Kurume University School of Medicine), Miwa Kawanaka (Department of Gastroenterology and Hepatology, Okayama University Graduate School of Medicine), Hirokazu Takahashi (Division of Metabolism and Endocrinology, Faculty of Medicine, Saga University), Nobuharu Tamaki (Department of Gastroenterology and Hepatology, Musashino Red Cross Hospital), Hayato Nakagawa (Department of Gastroenterology and Hepatology, Graduate School of Medicine, Mie University), Hayato Hikita (The University of Osaka, Graduate School of Medicine), Hideki Fujii (Department of Hepatology, Graduate School of Medicine, Osaka Metropolitan University), Kanji Yamaguchi (Molecular Gastroenterology and Hepatology, Graduate School of Medical Science, Kyoto Prefectural University of Medicine), Masato Yoneda (Department of Gastroenterology and Hepatology, Yokohama City University Graduate School of Medicine).

Collaborator: Kazuyoshi Kon (Department of Gastroenterology, Juntendo University Graduate School of Medicine), Akira Uchiyama (Department of Gastroenterology, Juntendo University Graduate School of Medicine), Yuya Seko (Molecular Gastroenterology and Hepatology, Graduate School of Medical Science, Kyoto Prefectural University of Medicine), Sadatsugu Sakane (The University of Osaka, Graduate School of Medicine), Ryuta Shigefuku (Department of Gastroenterology and Hepatology, Graduate School of Medicine, Mie University), Naoto Fujiwara (Department of Gastroenterology and Hepatology, Graduate School of Medicine, Mie University), Michihiro Iwaki (Department of Gastroenterology and Hepatology, Yokohama City University Graduate School of Medicine), Takashi Kobayashi (Department of Gastroenterology and Hepatology, Yokohama City University Graduate School of Medicine), Takuya Adachi (Department of Gastroenterology and Hepatology, Academic Fields of Medicine, Dentistry, and Pharmaceutical Sciences, Okayama University), Yasuto Takeuchi (Department of Gastroenterology and Hepatology, Academic Fields of Medicine, Dentistry, and Pharmaceutical Sciences, Okayama University), Tsubasa Tsutsumi (Division of Gastroenterology, Department of Medicine, Kurume University School of Medicine), Dan Nakano (Division of Gastroenterology, Department of Medicine, Kurume University School of Medicine), Kaoru Shibayama (Department of Comprehensive & Fundamental Nursing, Saga University), Noriyo Urata (Department of General Internal Medicine 2, General Medical Center, Kawasaki Medical School), Hisamitsu Miyaaki (Department of Gastroenterology and Hepatology, Graduate School of Biomedical Sciences, Nagasaki University), Hidekatsu Kuroda (Division of Gastroenterology and Hepatology, Department of Internal Medicine, Iwate Medical University School of Medicine), Masahiro Koseki (Department of Cardiovascular Medicine, The University of Osaka Graduate School of Medicine), Hirohito Sone (Department of Hematology, Endocrinology and Metabolism, Faculty of Medicine, Niigata University), Yasuhiro Matsubayashi (Department of Hematology, Endocrinology and Metabolism, Faculty of Medicine, Niigata University).

### Evaluation committee

Chair: Yoshiyuki Takei, Vice-chair: Masataka Seike (Oita Cardiovascular Hospital), Keisuke Kakisaka (Division of Gastroenterology and Hepatology, Department of Internal Medicine, School of Medicine, Iwate Medical University), Atsushi Takai (Department of Gastroenterology and Hepatology, Graduate School of Medicine, Kyoto University), Kazuo Notsumata (Municipal Tsuruga Hospital).

### The Japanese Society of Gastroenterology (JSGE)

Director General: Satoshi Mochida (Saitama Medical University Hospital); Past Director General: Kazuhiko Koike (Kanto Central Hospital [Kanto Central Hospital of the Mutual Aid Association of Public School Teachers]); Vice Director General: Susumu Eguchi (Nagasaki University Hospital), Past Vice Director General: Yoshifumi Takeyama (Osaka Gyoumeikan Hospital); Director of the Guidelines Editorial Committee: Takao Itoi (Tokyo Medical University Hospital); Vice Director of the Guidelines Editorial Committee: Hajime Isomoto (Tottori University Hospital); Chairperson of the Guidelines Editorial Committee: Sumio Watanabe (Juntendo University Hospital); Members of the Guidelines Editorial Committee: Mitsuo Shimada (Saito Clinic), Shinsaku Fukuda (Hirosaki University Hospital), Susumu Tazuma (Futabanosato Hospital).

### The Japan Society of Hepatology (JSH)

Director General: Tetsuo Takehara (Kansai Rosai Hospital); Chair of the Guidelines Steering Committee: Hiroshi Yotsuyanagi (National Center for Global Health and Medicine); Members of the Guidelines Steering Committee: Naoya Sakamoto (Hokkaido University Hospital), Masayuki Kurosaki (Japanese Red Cross Musashino Hospital), Tsuyoshi Ishikawa (Yamaguchi University Hospital), Masahito Shimizu (Gifu University Hospital), Gouki Suda (Hokkaido University Hospital), Ryosuke Tateishi (The University of Tokyo Hospital), Makiko Taniai (Tokyo Women's Medical University Hospital).
