# Supplementary material for: Evidence-based clinical practice guidelines for metabolic dysfunction-associated steatotic liver disease (MASLD) 2026
Source: J Gastroenterol. 2026 May 13;61(6):693–710. doi: 10.1007/s00535-026-02408-2 (PMC13219138; doi:10.1007/s00535-026-02408-2)
Supplement: Supplementary file 1 — Supplementary file1 (DOCX 26 KB) [file 535_2026_2408_MOESM1_ESM.docx]

**Chapter 1: Etiology and Natural Course**

**BQ. What is the prevalence of MASLD?**

- The prevalence of MASLD varies by country and region and is generally higher in men than in women.

**BQ. Is the risk of liver-related events increased in MASLD?**

- Patients with MASLD are at higher risk of liver-related events—including HCC, variceal bleeding, and liver failure—than the general population, and this risk increases with disease stage.

**BQ. Is there an increased risk of non-liver-related events in MASLD?**

- The risks of CVD events and CKD are increased in MASLD, as is the risk of extrahepatic malignancies.

**FRQ. Is there a difference in the risk of clinical events between MetALD and MASLD?**

- Compared with MASLD, MetALD is associated with a higher risk of liver-related events. By contrast, findings regarding cardiovascular risk in MetALD are inconsistent, with some studies reporting an increased risk and others reporting a risk comparable to that in MASLD; therefore, further investigation is required.

**FRQ. What are the challenges in determining the alcohol intake threshold for MetALD?**

- Although alcohol consumption exceeding MASLD criteria clearly contributes to liver injury, there is insufficient scientific evidence to define an upper limit of alcohol intake for MetALD, and this remains an important issue for future investigation.

**Chapter 2: Pathogenesis**

**BQ. Does alcohol affect the pathogenesis of MASLD?**

- Even low-to-moderate alcohol consumption may worsen the disease course of MASLD; therefore, habitual alcohol intake should, in principle, be avoided in patients with MASLD, even at low levels.

**FRQ. How do genetic factors contribute to MASLD development and progression?**

- Genetic polymorphisms in lipid metabolism and inflammatory pathways influence susceptibility to MASLD and disease severity.

**FRQ. What are the possible mechanisms of hepatocellular damage in MASLD?**

- In MASLD, abnormal lipid accumulation triggers a complex cascade of pathogenic processes, including lipotoxicity, oxidative stress, mitochondrial dysfunction, endoplasmic reticulum stress, and immune responses, leading to hepatocellular injury. The full spectrum of these mechanisms has not yet been fully elucidated, and further research is required.

**FRQ. Do changes in the intestinal microbiota affect the pathogenesis of MASLD?**

- Alterations in the gut microbiota are thought to influence the pathophysiology of MASLD. Dysbiosis, in particular, has been suggested to be associated with progression of hepatic inflammation and fibrosis; however, further studies are required to clarify causal relationships in humans.

**FRQ. Do organs other than the liver affect the pathogenesis of MASLD?**

- Organs other than the liver, including adipose tissue, pancreas, skeletal muscle, the nervous system, and the intestinal tract, contribute to the pathogenesis of MASLD.

**FRQ. What are the possible mechanisms of hepatocarcinogenesis in MASLD?**

- Hepatocarcinogenesis arises from complex interactions initiated by dysregulated lipid metabolism and oxidative stress, followed by chronic inflammation, insulin resistance, disruption of the immune microenvironment, alterations in the gut environment, and genetic factors.

**FRQ. How does the intrahepatic microenvironment affect the pathogenesis of MASLD?**

- In MASLD, interactions among intrahepatic cell populations—including hepatocytes, hepatic stellate cells, macrophages, and hepatic sinusoidal endothelial cells—play a central role in the development of liver pathology.

**Chapter 3: Comorbidities**

**BQ. What is the relationship between MASLD and T2DM?**

- MASLD and T2DM are closely associated and exhibit a bidirectional relationship, mutually influencing disease onset, disease progression, event occurrence, and patient prognosis.

**BQ. What is the relationship between MASLD and obesity?**

- Obesity and its severity are associated with disease progression in MASLD.

**BQ. What is the relationship between MASLD and dyslipidemia?**

- MASLD is frequently associated with dyslipidemia. While progression of MASLD affects lipid profiles, worsening dyslipidemia may also contribute to MASLD progression.

**BQ. What is the relationship between MASLD and hypertension?**

- The prevalence of hypertension is higher in patients with MASLD than in the general population. Hypertension is also an independent contributing factor to liver fibrosis progression in MASLD.

**BQ. Is there a relationship between MASLD and sleep apnea syndrome, endocrine disorders, and hyperuricemia?**

- MASLD is associated with sleep apnea syndrome, various endocrine disorders, and hyperuricemia.

**BQ. Is MASLD associated with CKD?**

- MASLD is associated with an increased risk of CKD and may adversely affect prognosis. However, because MASLD and CKD share common risk factors, it remains unclear whether a direct causal relationship exists.

**BQ. Is MASLD associated with the risk of non-hepatic malignancies?**

- MASLD is associated with an increased risk of colorectal cancer and has also been linked to higher risks of gastric, esophageal, and gynecological cancers.

**FRQ. Is liver pathology in MASLD associated with the risk of cerebrovascular disease?**

- MASLD itself increases the risk of cardiovascular and cerebrovascular disease; however, it remains unclear whether liver fibrosis independently contributes to this risk.

**FRQ. What is the risk of cardiovascular events in Japanese patients based on CMRFs associated with MASLD?**

- In Japanese patients with MASLD, cardiovascular risk varies according to the number of concomitant CMRFs and the degree of improvement in individual CMRF components. Among these factors, diabetes mellitus appears to have a particularly strong impact on CVD risk.
- In MASLD, CMRFs are defined based on conventional international criteria for MetS; however, further validation of the appropriateness of each cutoff value and component is required in patients with steatotic liver disease.

**Chapter 4: Diagnosis**

**Invasive tests**

**BQ. Is a liver biopsy required for the diagnosis of MASLD?**

- Liver biopsy is not mandatory for the diagnosis of MASLD. With recent advances in noninvasive diagnostic methods, disease severity—particularly the degree of liver fibrosis—can now be assessed with reasonable accuracy without biopsy. However, liver biopsy remains valuable in selected situations, such as establishing a definitive diagnosis of at-risk MASH, assessing inflammatory activity, resolving discrepancies between noninvasive tests, and differentiating MASLD from other chronic liver diseases.

**Noninvasive tests**

**CQ. Are biomarkers and scoring systems useful for diagnosing and assessing fibrosis and inflammation in MASLD?**

- Liver fibrosis and inflammation in MASLD are closely related to prognosis. Hematological biomarkers and scoring systems are useful for assessing fibrosis progression, inflammatory activity, and at-risk MASH; therefore, their use is recommended. (Evidence Level A, strong recommendation)

**CQ. Are noninvasive steatosis diagnostic methods useful for diagnosing SLD?**

- Noninvasive diagnostic methods for SLD, including ultrasound B-mode imaging, MRI–proton density fat fraction (MRI-PDFF), and ultrasound attenuation methods, are useful and therefore recommended. (Evidence Level A, strong recommendation)

**CQ. Is hepatic fat quantification useful for predicting progression of liver disease?**

- Because hepatic fat content and its longitudinal changes may be associated with disease progression, serial assessment of hepatic fat is suggested. (Evidence Level C, weak recommendation)

**CQ. Is ultrasound elastography useful for diagnosing fibrosis in MASLD?**

- Ultrasound elastography is recommended for assessing fibrosis in MASLD. (Evidence Level A, strong recommendation)

**CQ. Is MRE useful for diagnosing fibrosis in MASLD?**

- MRE is useful for diagnosing fibrosis in MASLD and is recommended as a noninvasive diagnostic method at facilities where it is available. (Evidence Level A, strong recommendation)

**FRQ. Are combination scores that integrate liver stiffness with multiple factors useful in the clinical management of MASLD?**

- Combination scores such as FAST, MAST, MEFIB, and Agile are useful for risk stratification and determining therapeutic intervention. Each score should be used appropriately according to its characteristics and the clinical setting.

**Consultation standards**

**BQ. What are the criteria for gastroenterological consultation in patients with MASLD?**

- Consultation with a gastroenterologist/hepatologist is recommended for patients with advanced or suspected liver fibrosis, low platelet count, persistently elevated AST or ALT levels, or imaging findings suggestive of cirrhosis.

**Others**

**FRQ. Is AI useful for diagnosing MASLD?**

- AI may be useful for diagnosing MASLD, predicting prognosis, improving reproducibility of pathological assessment, and supporting diagnostic imaging; however, further validation is required.

**Chapter 5: Follow-up**

**FRQ. What is the appropriate screening method for liver cirrhosis and liver cancer due to MASLD?**

- In MASLD, risk stratification based on the progression of liver fibrosis is essential. In principle, HCC screening every 6 months is recommended for patients with advanced liver fibrosis. However, screening strategies for patients without advanced fibrosis have not yet been established and remain an important area for future research. Regular assessment of liver fibrosis during follow-up is also necessary because it may facilitate early detection of cirrhosis and liver failure.

**FRQ. What are the criteria for cerebrovascular disease screening and specialist consultation in patients with MASLD?**

- Screening methods for cardiovascular and cerebrovascular diseases specifically tailored to MASLD, as well as clear criteria for consultation with relevant specialists, have not yet been established. Therefore, assessment of risk factors using existing screening approaches and appropriate specialist consultation are required.

**FRQ. Is screening for malignant diseases other than those related to the liver necessary in patients with MASLD?**

- Screening for extrahepatic malignancies, such as colorectal cancer, is suggested according to age. However, the optimal screening modalities and intervals have not been sufficiently validated.

**FRQ. What is the role of intervention in MASLD from a medical economic perspective?**

- Non-invasive screening of high-risk groups to identify cases of advanced fibrosis is cost-effective.

**Chapter 6: Treatment**

**Lifestyle modifications**

**BQ. What is the role of dietary therapy in MASLD?**

- Weight loss, reduction in dietary energy intake, and appropriate macronutrient composition are beneficial for the improvement of MASLD. In particular, the Mediterranean diet has been associated with a reduction in hepatic fat content. In addition, recent studies have suggested potential benefits of the Japanese diet in patients with MASLD.

**BQ. What is the role of exercise therapy in MASLD?**

- Exercise therapy is effective not only for reducing intrahepatic fat mass in patients with MASLD, but also for improving glucose and lipid metabolism disorders and mitigating sarcopenia. Thus, exercise is considered a fundamental treatment.

**BQ. What percentage of weight loss is effective in MASLD?**

- Weight loss of 5% or more is considered effective for achieving histological improvement; however, in non-obese patients, weight loss of approximately 3–5% may be effective.

**FRQ. Is behavioral therapy effective for MASLD?**

- Behavioral therapy aimed at lifestyle improvement through multidisciplinary collaboration and digital tools is useful; however, evidence supporting the effects of behavioral therapy on liver-related outcomes in MASLD remains insufficient.

**FRQ. What are the prospects for liver rehabilitation in MASLD?**

- In MASLD, not only obesity but also the coexistence of sarcopenia is frequently observed. Implementation of liver-focused rehabilitation may help prevent MASLD and cardiovascular disease and is expected to improve quality of life.

**Drug therapy**

**BQ. Are liver-protective agents effective for MASLD?**

- There is insufficient evidence to support the efficacy of ursodeoxycholic acid, glycyrrhizin preparations, or other hepatoprotective agents for the treatment of MASLD; therefore, their use is not recommended.

**CQ. Are GLP-1RA effective for MASLD?**

- The use of GLP-1RA is suggested for the treatment of MASLD. However, as of September 2025, these agents were not covered by insurance for MASLD. (Evidence Level A, weak recommendation)

**CQ. Are GIP/GLP-1RA effective for MASLD?**

- The use of GIP/GLP-1RA is suggested for the treatment of MASLD. However, as of June 2025, these agents were not covered by insurance for MASLD. (Evidence Level B, weak recommendation)

**CQ. Are SGLT2 inhibitors effective for MASLD?**

- SGLT2 inhibitors have been shown to improve hepatic steatosis, MASH, and liver fibrosis, and their use is suggested for the management of MASLD. However, evidence for histological improvement and long-term liver-related outcomes remains limited, and further studies are needed. These agents are not covered by insurance for MASLD. (Evidence Level B, weak recommendation)

**CQ. Is pioglitazone** **effective for MASLD?**

- Pioglitazone has been shown to improve liver histology and liver enzyme abnormalities in patients with MASLD, and its use is suggested. However, treatment should be undertaken with caution because of potential adverse effects. Pioglitazone is not covered by insurance for MASLD. (Evidence Level B, weak recommendation)

**CQ. Are lipid metabolism-modifying agents effective for MASLD?**

Pemafibrate

- There is no evidence that pemafibrate improves liver histology in patients with MASLD and concomitant hypertriglyceridemia. However, improvements in liver enzyme abnormalities have been reported, and its use is suggested. Pemafibrate is not covered by insurance for the treatment of MASLD. (Evidence Level C, weak recommendation)

Statins

- Although statins have not been shown to clearly improve liver histology in patients with MASLD and concomitant hypercholesterolemia, they improve liver enzyme abnormalities and liver-related outcomes; therefore, their use is suggested. Statins are not covered by insurance for the treatment of MASLD. (Evidence Level C, weak recommendation)

**CQ. Is vitamin E effective for MASLD?**

- Vitamin E has been shown to improve liver histology and liver enzyme abnormalities in patients with MASLD. However, its overall effectiveness is limited, and its use should therefore be considered on a case-by-case basis. Vitamin E is not covered by insurance for the treatment of MASLD. (Evidence Level B, weak recommendation)

**FRQ. Are other diabetes medications effective for MASLD?**

- At present, there are no other antidiabetic agents with sufficient evidence to support their efficacy for the treatment of MASLD.

**FRQ. Is resmetirom effective for MASH?**

- Resmetirom has demonstrated efficacy in the treatment of MASH in patients with fibrosis stages F2–F3. However, resmetirom is not currently approved for use in Japan.

**FRQ. What are the promising drugs for MASLD in the future?**

- In the future, pharmacotherapies centered on thyroid hormone receptor-β agonists, GLP-1RA, PPAR agonists, and fibroblast growth factor 21 analogues are expected to be promising for the treatment of MASLD. Furthermore, advances in combination therapies and gene-targeted treatments are anticipated to enable suppression of disease progression and the realization of personalized medicine.

**FRQ. What are the challenges in endpoints for MASLD treatment?**

- In MASLD treatment, improvement in fibrosis and resolution of MASH are commonly used endpoints; however, it remains unclear whether these endpoints translate into improved prognosis.

**Surgical treatment**

**BQ. Is metabolic and bariatric surgery effective for MASLD?**

- In patients with MASLD and severe obesity, metabolic and bariatric surgery is an effective therapeutic option for improving liver disease. However, comprehensive treatment planning is essential, including careful consideration of surgical indications in patients with liver cirrhosis and the risk of postoperative MASLD recurrence.

**BQ. What are the characteristics of liver transplantation for decompensated cirrhosis due to MASLD?**

- Liver transplantation for MASLD-related decompensated cirrhosis is increasing. Post-transplant survival is comparable to that of liver transplantation for other liver diseases; however, the incidence of comorbidities, including cardiovascular disease, is higher.

**Others**

**FRQ. Is coffee consumption effective for MASLD?**

- Multiple meta-analyses have shown that habitual coffee consumption is associated with improvement in liver fibrosis in patients with MASLD assessed by noninvasive tests. However, efficacy has not been demonstrated in randomized controlled trials, and the specific responsible components remain unclear.

**FRQ. Are probiotics useful for MASLD?**

- Probiotics may be beneficial for improving the pathophysiology of MASLD.
